# Supplementary material for: Comparative multiomics analysis of cell physiological state after culture in a basket bioreactor
Source: Sci Rep. 2022 Nov 23;12:20161. doi: 10.1038/s41598-022-24687-4 (PMC9686226; doi:10.1038/s41598-022-24687-4)
Supplement: Supplementary file 1 — Supplementary Information 1. [file 41598_2022_24687_MOESM1_ESM.zip › raw data/Proteomic raw data/6.KEGGenrichment/EG.vs.CG/DOWN/pathway/src/04071.html]

map04071


- K12348, K12349, K01441

- Down regulated proteins

A0A0D9RVZ2(Description:Ceramidase;Gene:ASAH1;log2FC:-1.3699)

- K01379

- Down regulated proteins

A0A0D9RBZ8(Description:Cathepsin D;Gene:;log2FC:-1.4696)

- K12350

- Down regulated proteins

A0A0D9QWC2(Description:Sphingomyelin phosphodiesterase;Gene:SMPD1;log2FC:-1.6416)

Close
